# Parametric Imaging of [^11^C]Flumazenil Binding in the Rat Brain

**DOI:** 10.1007/s11307-017-1098-2

**Published:** 2017-06-19

**Authors:** Isadora Lopes Alves, David Vállez Vállez García, Andrea Parente, Janine Doorduin, Ana Maria Marques da Silva, Michel Koole, Rudi Dierckx, Antoon Willemsen, Ronald Boellaard

**Affiliations:** 1 Department of Nuclear Medicine and Molecular Imaging, University Medical Center Groningen, University of Groningen, Groningen, The Netherlands; 2 Laboratory of Medical Imaging, School of Physics, Pontifícia Universidade Católica do Rio Grande do Sul, Porto Alegre, Brazil; 3 Department of Nuclear Medicine and Molecular Imaging, KU Leuven, Leuven, Belgium

**Keywords:** [^11^C]flumazenil, Rat, Parametric imaging, Small-animal imaging, PET

## Abstract

**Purpose:**

This study evaluates the performance of several parametric methods for assessing [^11^C]flumazenil binding distribution in the rat brain.

**Procedures:**

Dynamic (60 min) positron emission tomography data with metabolite-corrected plasma input function were retrospectively analyzed (male Wistar rats, *n* = 10). Distribution volume (*V*_T_) images were generated from basis function method (BFM), Logan graphical analysis (Logan), and spectral analysis (SA). Using the pons as pseudo-reference tissue, binding potential (*BP*_ND_ and DVR–1) images were obtained from receptor parametric imaging algorithms (RPM and SRTM2) and reference Logan (RLogan). Standardized uptake value images (SUV and SUVR) were also computed for different intervals post-injection. Next, regional averages were extracted from the parametric images, using pre-defined volumes of interest, which were also applied to the regional time-activity curves from the dynamic data. Parametric data were compared to their regional counterparts and to two-tissue compartment model (2TCM)-based values (previously defined as the model of choice for rats). Parameter agreement was assessed by linear regression analysis and Bland-Altman plots.

**Results:**

All parametric methods strongly correlated to their regional counterparts (*R* ^2^ > 0.97) and to the 2TCM values (*R* ^2^ ≥ 0.95). SA and RLogan underestimated *V*_T_ and *BP*_ND_ (slope of 0.93 and 0.86, respectively), while SUVR-1 overestimated *BP*_ND_ (slope higher than 1.07 for all intervals). While BFM and SRTM2 had the smallest bias to 2TCM values (0.05 for both), ratio Bland-Altman plots showed Logan and RLogan displayed relative errors which were comparable between different regions, in contrast with the other methods. Although SUV consistently underestimated *V*_T_, the bias in this method was also constant across regions.

**Conclusions:**

All parametric methods performed well for the analysis of [^11^C]flumazenil distribution and binding in the rat brain. However, Logan and RLogan slightly outperformed the other methods in terms of precision, providing robust parameter estimation and constant bias. Yet, other methods can be of interest, because they can provide tissue perfusion (*i*.*e*., *K*_1_ with BFM and SA), relative flow (*i*.*e*., *R*_1_ with RPM and SRTM2), and model order (SA) images.

## Introduction

The *in vivo* study of neuronal integrity has been well established by means of positron emission tomography (PET) imaging with [^11^C]flumazenil [1, 2]. A number of different conditions have previously been studied with [^11^C]flumazenil [3–5], and the link between the GABA-ergic system and neurological disorders [6] and inflammation [7] has increased the applicability and interest in PET imaging with this radiotracer.

In addition to the non-invasive character of PET, one of its main advantages for studying physiological processes is that it can provide quantitative information. Full quantitative analysis can be achieved by pharmacokinetic modeling, which describes the time course of the PET tracer in tissue and enables the estimation of different parameters related to tracer distribution, metabolism, or receptor density and binding [8, 9]. In that context, the quantification of [^11^C]flumazenil binding has been previously performed by different pharmacokinetic models. In human studies, the one-tissue compartment model (1TCM) and the simplified reference tissue model (SRTM) [10] using the pons as a reference tissue have been validated [11] and applied [12–14]. Although useful, these models have been mostly applied for regional quantification of [^11^C]flumazenil binding, analyzing the average kinetic profile of a specific volume of interest (VOI).

In cases when subtle or disease-specific changes are expected, VOI-based pharmacokinetic modeling might be suboptimal precisely due to the use of pre-defined VOIs. If physiological changes are restricted to a subset of a region (tissue heterogeneity) or if they do not follow anatomical delineations, the average signal from pre-defined VOIs might not be able to properly describe the underlying alterations. There, the generation and analysis of parametric images can be of greater use. Parametric images are graphical representations of quantitative endpoints, where every image voxel corresponds to a kinetic parameter, such as distribution volume (*V*_T_) or binding potential (*BP*_ND_). Although parametric imaging is most commonly applied to human studies, it could be of great interest for the analysis of animal PET data. Preclinical PET imaging plays an important role in drug development and treatment assessment studies, for example, and it could be advantageous to explore full kinetic analysis of subtle physiological changes by parametric imaging also in the context of such animal study designs. Moreover, parametric imaging enables group comparisons at the voxel level using statistical parametric mapping (SPM), which performs the statistical analysis independent of pre-defined regions of interest.

In human studies, parametric images of [^11^C]flumazenil binding have been generated from a number of different methods, and the Logan graphical analysis, a multilinear reference tissue model (MRTM2), and the receptor parametric mapping method (RPM) were found to provide the best quantitative metrics [15]. However, no similar analysis has been performed for animal data. Moreover, the preference and performance of VOI-based models have already been shown to differ between human and rat studies [16]. Together with the higher noise levels typical of animal PET data, this difference could indicate the parametric models found to work best for the analysis of human studies might not be the most appropriate in the pre-clinical setting.

The aim of the current study was, therefore, to investigate the performance of several parametric methods for the analysis of [^11^C]flumazenil PET images of the rat brain. To that end, we retrospectively analyzed [^11^C]flumazenil pre-clinical images with both plasma input and reference tissue-based parametric methods and compared the results to their corresponding VOI-based pharmacokinetic models.

## Material and Methods

### Animal Data

We retrospectively analyzed male Wistar-Unilever outbred rats (*n* = 10) obtained from Harlan (Horst, The Netherlands). The animal experiments were performed according to the Dutch Law on Animal Experiments and were approved by the Institutional Animal Care and Use Committee of the University of Groningen (6264B).

### Data Acquisition

Prior to the PET scan, a mixture of 5 % isoflurane and medical air was used to anesthetize the animals, which were maintained under anesthesia at 1.5–2.0 %, with a flow of 1.5–2 ml/min. Scans were performed in the Focus 220 camera (Siemens Medical Solutions, USA), where animals were positioned transaxially with the head in the field of view. First, a transmission scan was performed with a point-source of Co-57. Next, [^11^C]flumazenil was injected (bolus injection) with an automatic pump over 60s, and the PET camera was started at the moment of injection. List-mode PET data was acquired for a period of 60 min. [^11^C]Flumazenil was synthesized as previously described [17], and a 53 ± 18 % radiochemical yield was obtained, with radiochemical purity of [^11^C]flumazenil of 100 % and a pH of 6.5–7. The mean injected activity was 59.3 ± 22.6 MBq, with a 3.3 ± 2.4 nmol of injected mass, and the weight of the animals was 0.273 ± 0.03 kg [18].

During the scan, arterial blood samples (0.1 ml) were obtained (*n* = 16) for each individual animal, corresponding to 5, 10, 15, 30, 45, and 60 min post-injection (p.i.). The samples were subsequently separated into blood and plasma, and the activity was measured in a gamma counter (LKB-Wallac, Finland). For the metabolite analysis, 2–3 larger samples (0.6 ml) were collected for each animal, and a population metabolite curve was constructed by combining data from the individual samples. The validity of using a population-based metabolite curve in this animal group was previously assessed and reported elsewhere [17, 18]. Finally, this curve was used for the construction of individual metabolite-corrected plasma input functions.

### Image Processing

After all necessary corrections, the list-mode data was reconstructed into 21 frames (6 × 10, 4 × 30, 2 × 60, 1 × 120, 1 × 180, 4 × 300, and 3 × 600 s). The 2D–OSEM algorithm was used to reconstruct the Fourier rebinned sinograms, with 4 iterations and 16 subsets. The resulting images displayed a 128 × 128 × 95 matrix, 0.63 mm pixel width, and 0.79 mm slice thickness. Next, individual dynamic images were coregistered to a [^11^C]flumazenil template [19] in PMOD v3.7 (PMOD Technologies Ltd., Switzerland). Time-activity curves (TACs) were then generated from a set of pre-defined bilateral VOIs which included the whole brain, regions with high GABA_A_ expression (frontal cortex and hippocampus), and regions with low GABA_A_ expression (cerebellum, medulla, and pons). Following previous studies [16], the pons was set as the pseudo-reference tissue and its TAC was used as input for reference-based models. In order to minimize bias induced by noise in the parametric methods, an isotropic 3D Gaussian filter (FWHM = 0.8 mm) was applied. Finally, individually generated brain masks were also applied prior to modeling.

### Pharmacokinetic Modeling

All pharmacokinetic modeling was performed with the PPET [20] software package.

First, parametric *V*_T_ images were generated from three different methods, including the basis function method (BFM), which is an implementation of the 1TCM, the Logan graphical analysis (Logan) [21], and the spectral analysis (SA) [22]. For these methods, the metabolite-corrected plasma curve was used as input function, and blood volume fraction was accounted for as a fit parameter in BFM and SA. Next, parametric *BP*_ND_ images were computed from three reference-based models. The first was the reference tissue Logan graphical analysis (RLogan) [23], which provides the distribution volume ratio (DVR) of target regions relative to the reference tissue. Following the relationship *BP*_ND_ = DVR−1 [8], we subtracted 1 from the RLogan DVR to allow direct comparison to parametric images of *BP*_ND_. The other two methods consisted of two implementations of the receptor parametric mapping method (RPM and SRTM2) [24], which correspond to the two versions of the simplified reference tissue model (SRTM and SRTM2). For the SRTM2, the model fit was performed in two steps, where the median value of the reference tissue efflux rate constant (${k}_2^{\prime }$) obtained from all voxels outside the reference tissue in the first run was entered as a fixed parameter for the second run [25]. An overview of the settings used for the parametric methods and optimized for this group can be found in Table 1.

**Table 1:** Overview of settings for parametric methods

Method		Start *t* (min)	End *t* (min)	Basis start *t* (min)	Basis end *t* (min)	# basis functions
*V*_T_ (*K*_1_)	BFM	–	–	0.00083	0.016	50
	Logan	10	60	–	–	
	SA	–	–	0.00333	0.333	30
SUV	SUV_30–40_	30	40	–	–	
	SUV_40–50_	40	50	–	–	
	SUV_50–60_	50	60	–	–	
*BP*_ND_ (*R*_1_)	RLogan	10	60	–	–	
	RPM	–	–	0.01	0.2	50
	SRTM2	–	–	0.01	0.2	50
	SUVR_30–40_–1	30	40	–	–	
	SUVR_40–50_–1	40	50	–	–	
	SUVR_50–60_–1	50	60	–	–	

Parametric images of standardized uptake value (SUV) and SUV target to pons ratio (SUVR) were also generated for the intervals 30–40 min, 40–50 min, and 50–60 min post-injection and subsequently referred to by SUV_start-end_ and SUVR_start-end_. In order to compare SUVR and *BP*_ND_ images, 1 was subtracted from the derived SUVR values, assuming equilibrium was reached for those intervals.

### Method Evaluation

Results from each of the parametric methods were compared to (1) the corresponding regional (VOI) analysis using equivalent kinetic models but also to (2) reference values based on regional two-tissue compartment model (2TCM) fits. The 2TCM-derived parameters (*V*_T_ and DVR−1) were considered as reference values for method comparison. An overview of parametric methods, their corresponding VOI-models, parameter of interest, and equivalent 2TCM parameter can be found in Table 2.

**Table 2:** Overview of VOI-based and parametric methods and their corresponding quantitative parameters

VOI	Parametric	Parameter of interest	Additional parameter	Reference parameter
1TCM	BFM	*V*_T_	–	2TCM *V*_T_
Logan	Logan	*V*_T_	–	2TCM *V*_T_
SA	SA	*V*_T_	*K*_1_ and model order	2TCM *V*_T_
–	SUV	SUV	–	2TCM *V*_T_
RLogan	RLogan	DVR	–	2TCM DVR–1
SRTM	RPM	*BP*_ND_	*R*_1_	2TCM DVR–1
SRTM2	SRTM2	*BP*_ND_	*R*_1_	2TCM DVR–1
–	SUVR	SUVR	–	2TCM DVR–1

For that purpose, first the same pre-defined set of VOIs used for the generation of TACs were projected onto the parametric images, and average parameter estimates were obtained for each region. Next, the regional TACs were analyzed by six models corresponding to the parametric methods (*i*.*e*., 1TCM, Logan, SA, RLogan, SRTM, and SRTM2) and by the 2TCM. In the plasma input models, blood- and metabolite-corrected plasma curves were used as input function, with a fixed blood volume of 5 %. Finally, the regionally averaged values from parametric methods were compared to both their VOI-counterparts and to the corresponding 2TCM-derived reference.

### Statistical Analysis

Parameter agreement was assessed by linear regression analysis and Bland-Altman plots. Results were considered significant when *p* < 0.05 and are expressed as mean ± SD.

## Results

### Volume of Distribution

**Fig. 1:**
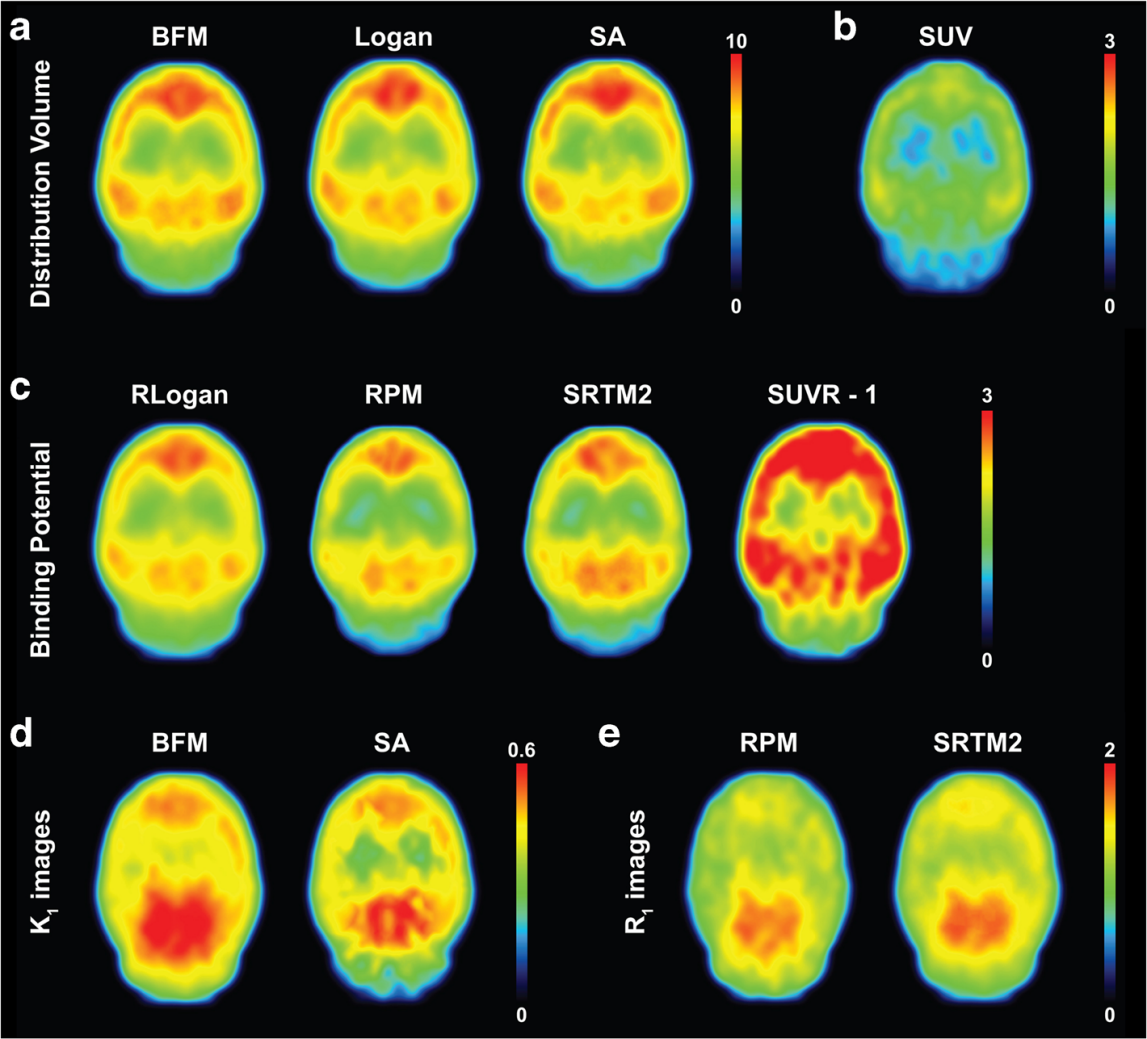
Representative (*n* = 1) parametric images of **a** *V*_T_, **b** SUV_30–40_, **c** *BP*_ND_, **d** *K*_1_ images, and **e** *R*_1_ images.

Visual inspection of parametric *V*_T_ images showed good correspondence between methods and the expected [^11^C]flumazenil distribution in the rat brain (Fig. 1).

**Fig. 2:**
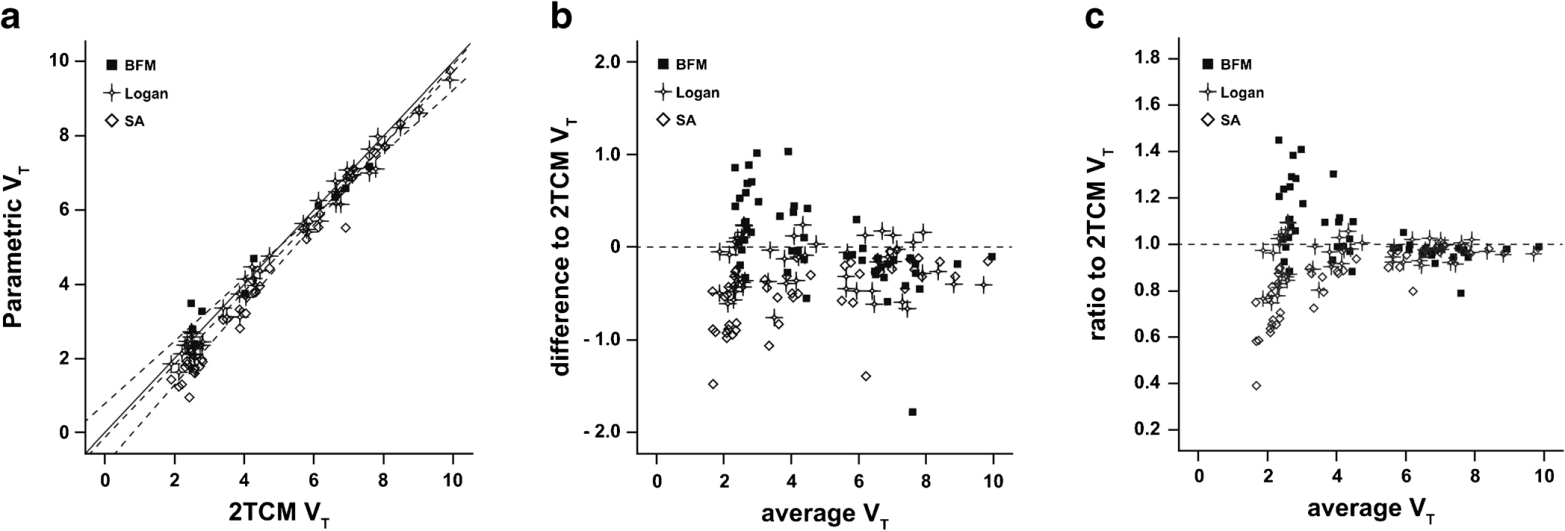
Regression analysis and Bland-Altman plots of *V*_T_ compared to 2TCM values. **a** Linear regression between parametric (BFM, Logan, and SA) and 2TCM *V*_T_ values determined by VOI analysis. The *solid line* is the identity line, and the *dashed lines* represent the regression lines. **b** Bland-Altman plot of the agreement in *V*_T_ estimation between the parametric methods and the reference values obtained from the 2TCM (VOI). The *dashed line* represents zero bias. **c** Ratio Bland-Altman plot between BFM, Logan, SA, and 2TCM *V*_T_ values. The *dashed line* represents a ratio of one, corresponding to full agreement between methods.

Compared to their VOI-counterpart, parametric Logan was the method with the best correlation, displaying an *R* ^2^ = 0.99 and a slope of 0.97 when the regression line was set through the origin (Table 3). The BFM also strongly correlated to its counterpart (1TCM) values (*R* ^2^ = 0.98), with a slope of 1.03. The slope of the linear regression of SA with its counterpart was further from identity (0.89), and the correlation was somewhat lower than the other methods (*R* ^2^ = 0.96). Overall, all three methods showed excellent correlation (*R* ^2^ = 0.99) to 2TCM reference *V*_T_ values (Fig. 2a). However, BFM was the method which displayed the slope closest to the identity line (0.98). Although SA showed the same correlation coefficient as the other two methods, a slope of 0.93 indicated a larger underestimation in *V*_T_ values.

**Table 3:** Linear regression analysis between parametric methods and their VOI-counterparts, as well as between parametric and reference values from 2TCM (VOI). Linear regression analysis was performed with and without setting the intercept to zero (through origin columns)

Model	VOI-counterpart				VOI-2TCM					
	*R* ^2^	Slope	Int.	*R* ^2^	Slope	*R* ^2^	Slope	Int.	*R* ^2^	Slope
				(Through origin)					(Through origin)	
Distribution volume										
BFM	0.96	0.79	1.35	0.98	1.03	0.96	0.85	0.78	0.99	0.98
Logan	0.99	1.03	−0.35	0.99	0.97	0.98	0.98	−0.13	0.99	0.96
SA	0.85	0.94	−0.28	0.96	0.89	0.98	1.08	−0.87	0.99	0.93
SUV_30–40_	–	–	–	–	–	0.87	0.27	−0.16	0.96	0.24
SUV_40–50_	–	–	–	–	–	0.87	0.21	−0.15	0.96	0.18
SUV_50–60_	–	–	–	–	–	0.83	0.16	−0.09	0.95	0.14
Binding potential										
RLogan	0.99	1.03	−0.10	0.99	0.97	0.98	0.92	−0.10	0.99	0.86
RPM	0.98	1.15	−0.09	0.99	1.08	0.97	0.98	−0.82	0.98	0.93
SRTM2	0.95	1.32	−0.63	0.97	0.91	0.97	0.89	0.15	0.98	0.98
SUVR_30–40_–1	–	–	–	–	–	0.98	1.21	−0.14	0.99	1.13
SUVR_40–50_–1	–	–	–	–	–	0.97	1.30	−0.16	0.98	1.21
SUVR_50–60_–1	–	–	–	–	–	0.94	1.17	−0.17	0.96	1.07

The Bland-Altman analysis showed that, despite the good correlation, a negative bias (−0.20) was present with the Logan method (Fig. 2b). The BFM had the smallest overall bias in relation to 2TCM *V*_T_ (0.05), yet its 95 % limits of agreement were slightly wider than those of Logan (Table 4). SA had the widest 95 % limits of agreement similar to BFM and an overall higher (negative) bias (−0.49). Figure 2c shows a ratio Bland-Altman plot to highlight the performance in parameter agreement for the different levels of *V*_T_ values. There, regions with high *V*_T_ (≥6) had a ratio close to one in comparison with 2TCM *V*_T_ values. On the other hand, larger over- and underestimations occurred for regions of lower *V*_T_ (<6).

**Table 4:** Bland-Altman analysis of agreement between parametric and VOI-counterpart methods, as well as between parametric and 2TCM (VOI) values

Parameter		VOI-counterpart		VOI-2TCM	
	Method	Bias	95 % L. A.	Bias	95 % L. A.
*V*_T_					
	BFM	0.44	−0.79 to 1.67	0.05	−0.89 to 1.00
	Logan	−0.20	−0.57 to 0.17	−0.19	−0.74 to 0.34
	SA	−0.57	−2.42 to 1.27	−0.49	−1.16 to 0.18
*BP*_ND_					
	RLogan	−0.07	−0.21 to 0.06	−0.17	−0.41 to 0.05
	RPM	0.03	−0.28 to 0.34	−0.10	−0.37 to 0.16
	SRTM2	−0.22	−0.74 to 0.29	0.05	−0.27 to 0.38
	SUVR_30–40_–1	–	–	0.05	−0.37 to 0.49
	SUVR_40–50_–1	–	–	0.13	−0.51 to 0.77
	SUVR_50–60_–1	–	–	−0.01	−0.59 to 0.58

**Fig. 3:**
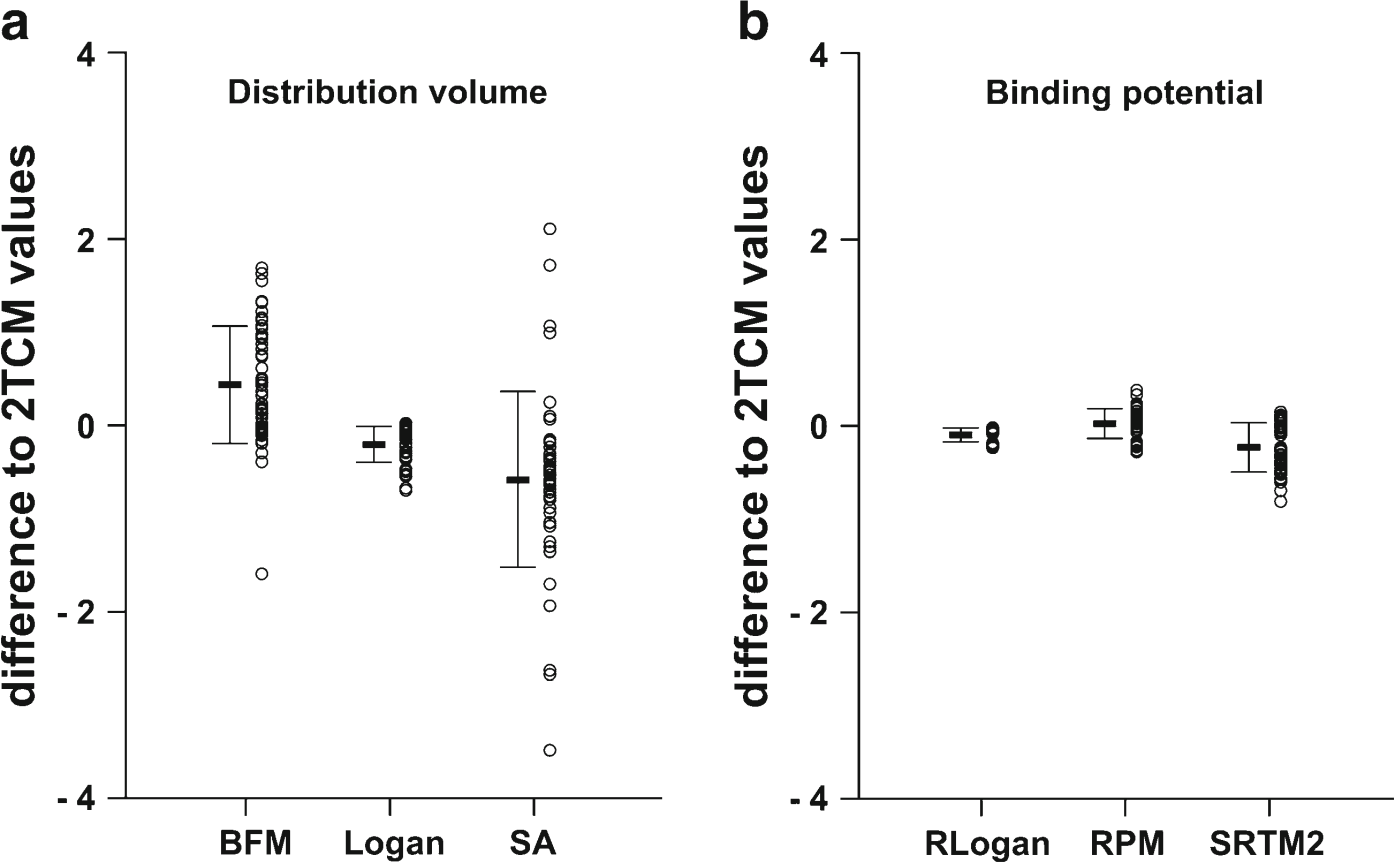
Plot of differences in estimates between 2TCM reference values across regions and **a** distribution volume and **b** binding potential. Individual values are represented by *circles*, the mean is represented by the *black line*, and the *bars* represent the SD.

Figure 3 displays the range of differences to 2TCM values for the three parametric methods across all regions. Significant differences in bias were found in all three methods when compared to 2TCM values. However, the bias from parametric Logan compared to 2TCM values showed the smallest variability (*SD* = 0.19), indicating higher precision for this method compared to the others.

### Binding Potential (*BP*_ND_ and DVR−1)

As can be seen in Fig. 1, the highest [^11^C]flumazenil binding spatially matched regions with a high receptor density (cortical and sub-cortical areas), while the lowest was seen in low-density ones (cerebellum and brainstem). Images of receptor binding were also visually comparable between parametric methods.

**Fig. 4:**
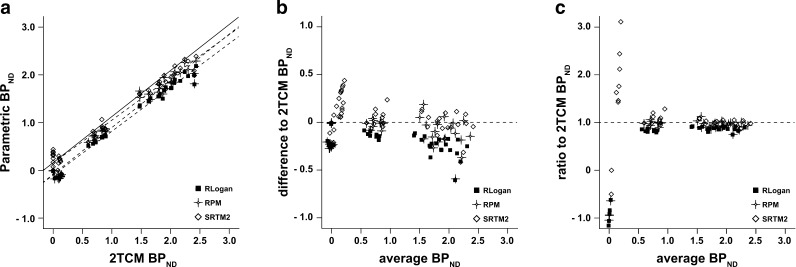
Regression analysis and Bland-Altman plots of *BP*_ND_. **a** Linear regression between parametric (RLogan, RPM, and SRTM2) and 2TCM DVR–1 (*BP*_ND_) values determined by VOI analysis. The *solid line* represents the identity line, and the *dashed lines* correspond to each of the regression lines. **b** Bland-Altman plot of the agreement in *BP*_ND_ estimation between the parametric methods and the reference values obtained from the 2TCM (VOI). The *dashed line* corresponds to zero bias. **c** Ratio Bland-Altman plot between *BP*_ND_ of parametric methods and 2TCM *BP*_ND_. The *dashed line* corresponds to a ratio of one, representing full agreement between methods. The *y*-axis is truncated at −1.0.

All reference-based methods performed well in comparison to 2TCM DVR−1 (Fig. 4a). In particular, DVR–1 from RLogan displayed the best correlation coefficient in relation to 2TCM values (*R* ^2^ = 0.99), while the slope was the furthest from the identity line (0.86), indicating underestimation of receptor binding. Binding estimates from RPM showed correlation to 2TCM values similar to those of RLogan (*R* ^2^ = 0.98) and a slope closer to identity (0.93) (Table 3). Estimation of binding from parametric SRTM2 outperformed both other methods in terms of linear regression, with a *R* ^2^ of 0.98 and a slope of 0.98 compared to 2TCM values.

Bland-Altman plots indicated a similar performance between RLogan and RPM methods (Fig. 4b). Both parametric methods displayed a negative bias compared to 2TCM DVR–1 (−0.17 for RLogan and −0.10 for RPM). SRTM2 showed a small overall bias (0.05) but resulted in the largest 95 % limits of agreement to 2TCM DVR–1, as can be seen in Table 4. For most levels of specific binding, all three methods showed good relative agreement, with a ratio close to one in comparison with 2TCM DVR–1 values (Fig. 4c). However, relative errors were larger for *BP*_ND_ values close to zero.

In Fig. 3, a similar performance between the reference methods can be seen. Significant differences were found in the bias of parametric RLogan and SRTM2 when compared to 2TCM values. No significant difference was seen for the RPM bias to 2TCM values (*p* = 0.20). Yet, RLogan displayed the highest precision (*SD* = 0.72) compared to the other two methods.

### SUV and SUVR–1

**Fig. 5:**
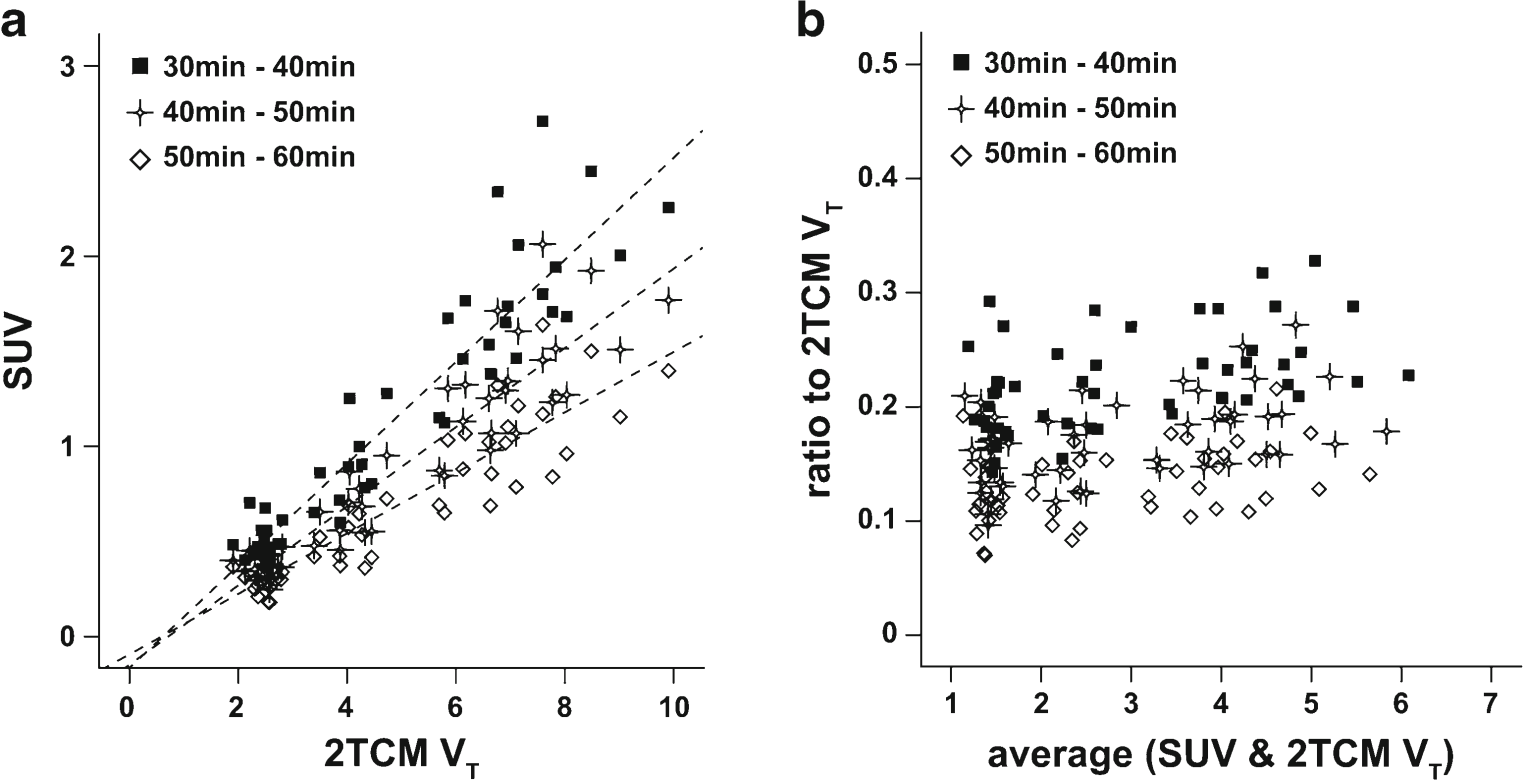
Regression analysis and ratio Bland-Altman plot of SUV from different intervals. **a** Regression analysis between SUV (parametric) and 2TCM *V*_T_ (VOI) for three different intervals p.i. The *dashed lines* represent the regression lines. **b** Ratio Bland-Altman plot between *V*_T_ estimates from SUV and from 2TCM *V*_T_ (VOI) for the same intervals.

Correlation between parametric SUV and 2TCM *V*_T_ was overall strong, with similar correlation coefficients across intervals p.i. (*R* ^2^ = 0.96, *R* ^2^ = 0.96, and *R* ^2^ = 0.95 for SUV_30–40_, SUV_40–50_, and SUV_50–60_, respectively). However, there was a clear scale difference between the two parameters, and a direct comparison was not possible, as SUV ranged from 0.2 to 2.7 and 2TCM *V*_T_ ranged from 1.9 to 9.9 (Fig. 5a). However, ratio Bland-Altman plots showed that SUV from all three intervals underestimated 2TCM *V*_T_ in a similar manner across regions, independent of the levels of uptake (Fig. 5b).

**Fig. 6:**
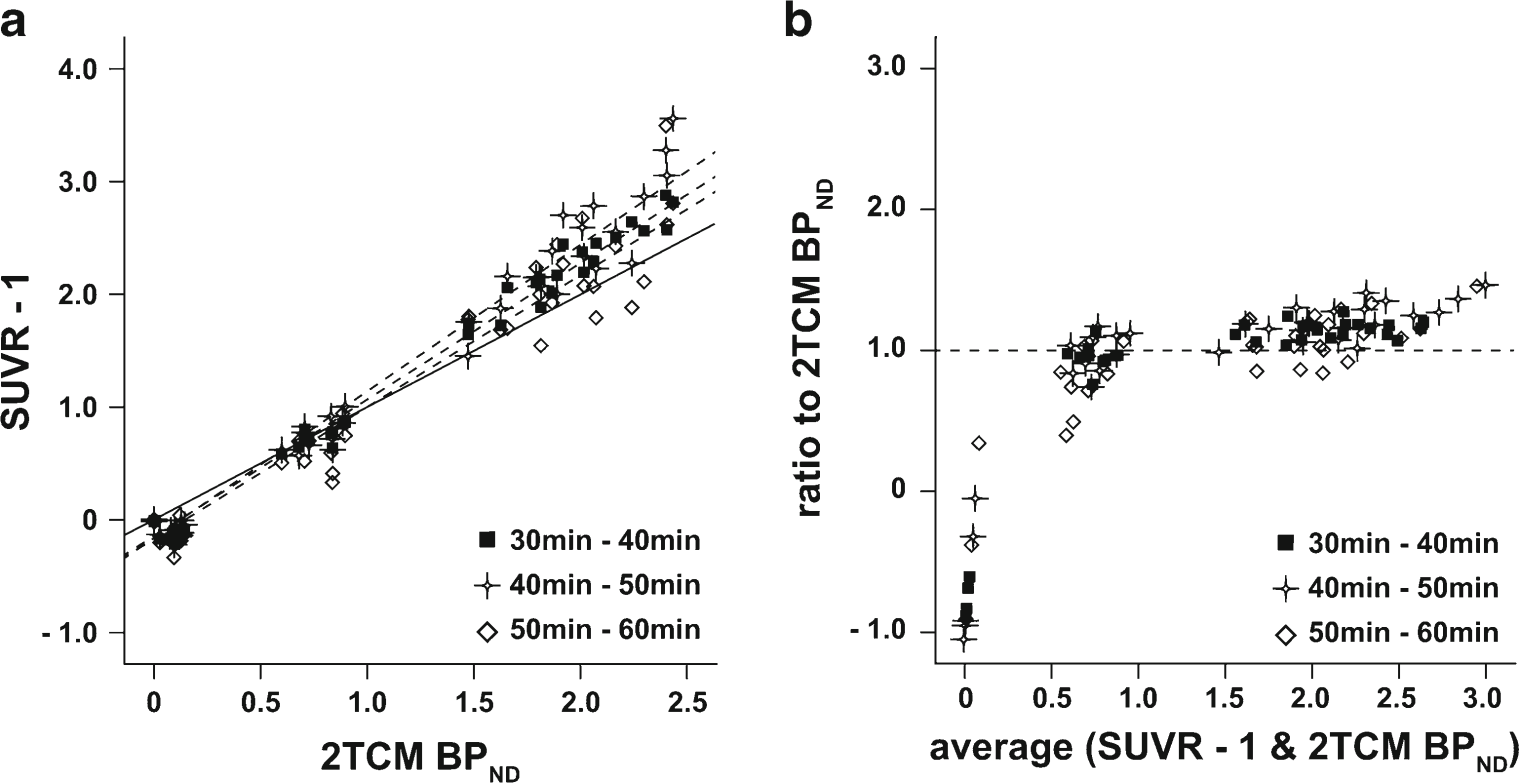
Regression analysis and ratio Bland-Altman plot of SUVR from different intervals. **a** Regression analysis between SUVR–1 (parametric) and 2TCM *BP*_ND_ (VOI) for three different p.i. intervals. The *solid line* represents the identity line, and the *dashed lines* correspond to each of the regression lines. **b** Ratio Bland-Altman plot between binding potential estimates from SUVR–1 and from 2TCM *BP*_ND_ (VOI) for the same intervals. The *dashed line* corresponds to a ratio of one, representing full agreement between methods. The *y*-axis is truncated at −1.0.

SUVR–1 images showed excellent correlation to binding estimates from 2TCM for all intervals p.i. (Fig. 6a). However, an overestimation of binding potential from SUVR–1 images was seen, with linear regression (through origin) slopes of 1.13, 1.21, and 1.07 for the SUVR_30–40_–1, SUVR_40–50_–1, and SUVR_50–60_–1, respectively. This was confirmed by a Bland-Altman analysis, where SUVR_40–50_–1 showed the highest bias compared to 2TCM DVR–1 and the largest 95 % limits of agreement (Table 4). In a ratio Bland-Altman, all intervals show similar performance, with the ratio close to one for most levels of specific binding (Fig. 6b). For binding values close to zero, relative errors were more pronounced, independent of the SUVR interval.

### Parametric Images of *K*_1_, *R*_1_, and Model Order

**Fig. 7:**
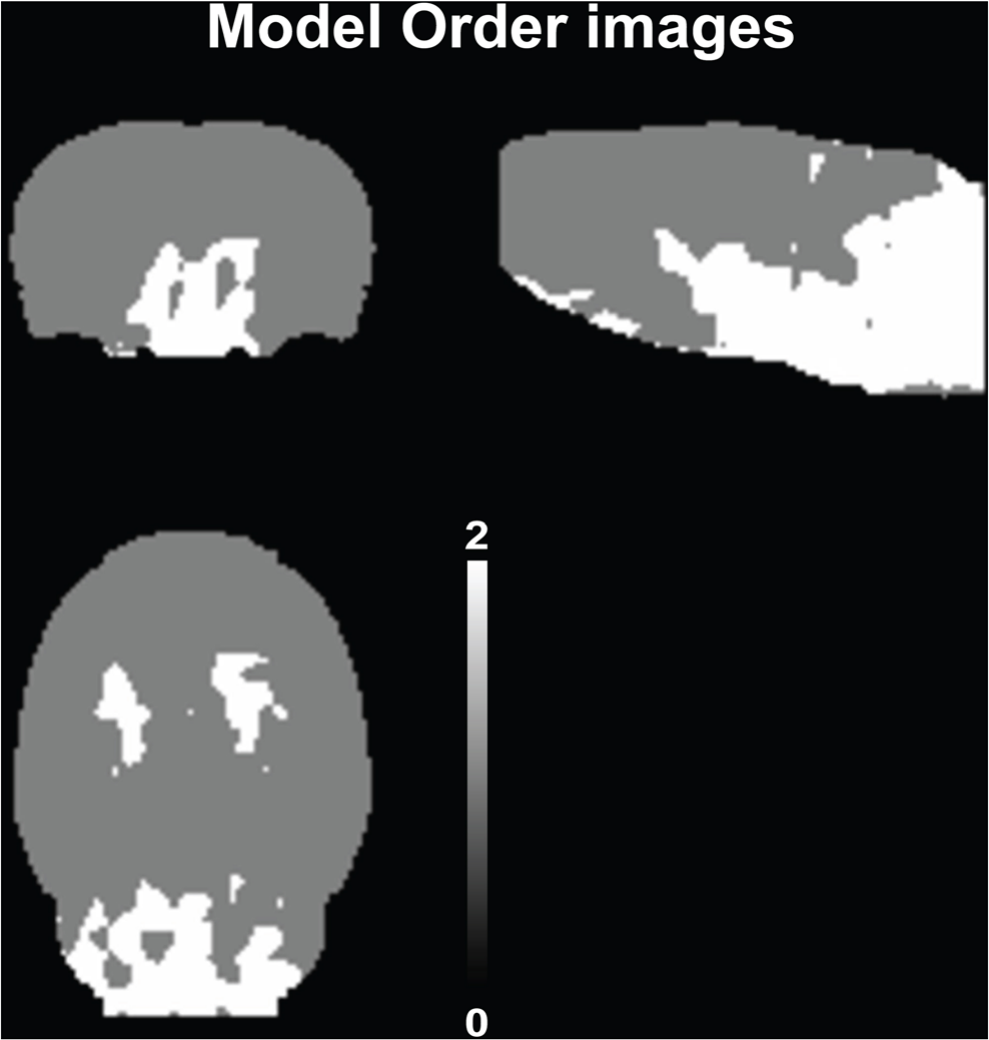
Representative image (*n* = 1) of model order obtained from the parametric SA method, displaying, in the different views, a difference between cortical and non-cortical regions.

For BFM and SA, parametric images of *K*_1_ (influx rate constant) were also available (Fig. 1). Although both displayed similar perfusion, BFM *K*_1_ was generally higher, while the less homogeneous distribution seen in the SA image suggested higher noise sensitivity for that method. SA also provided parametric images of model order, displaying the difference in the complexity of kinetic profiles between different voxels (Fig. 7). In those, a marked difference between high- and low-density regions is seen, and a more complex model was more frequently observed in the later, compared to the former.

Additional parametric images were also available from RPM and SRTM2 displaying *R*_1_, *i*.*e*., the ratio between *K*_1_ of a target region and of the pseudo-reference tissue (pons). As can be seen in Fig. 1, there is a good correspondence between the two methods. However, *R*_1_ images from SRTM2 displayed better contrast and lower noise levels compared to RPM *R*_1_ images.

Significant (*p* < 0.01) within-animal correlations were found between *K*_1_ and *R*_1_ values of parametric and VOI-counterparts for all animals when pooling the models (Spearman’s rho ranged from 0.57 to 0.80). However, when comparing parametric values to reference 2TCM estimates, significant within-animal correlations were found for only 7 out of 10 animals. Moreover, by grouping the animals and comparing estimates by model, only BFM *K*_1_ values were significantly correlated to VOI-counterpart estimates (Spearman’s rho 0.68, *p* < 0.01).

## Discussion

The use of parametric imaging methods for the analysis of PET data can be of importance when subtle and/or disease-specific changes are expected. In the case of [^11^C]flumazenil brain PET, those changes are related to the GABA-ergic system and can be present in several conditions related to neuronal loss and inflammatory processes. While parametric images of [^11^C]flumazenil distribution and binding have been generated and applied in human studies, the same has not yet been done for the pre-clinical setting. Therefore, this study evaluated several parametric methods for the visualization and analysis of [^11^C]flumazenil PET images of the rat brain.

A first analysis of parametric *V*_T_ images demonstrated that all three methods tested in this study (BFM, Logan, and SA) corresponded well to their VOI-counterparts. These results indicate that the generation of parametric *V*_T_ maps from these methods could, to some extent, replicate results obtained from a regional analysis, which is generally less affected by noise and image resolution. More importantly, there was also an excellent correlation between parametric values of each of the three parametric methods and *V*_T_ estimated from the reference 2TCM VOI analysis. In terms of parameter agreement, however, a slight difference between the methods becomes noticeable. While BFM *V*_T_ showed a small bias compared to 2TCM values (0.05), the wide 95 % limits of agreement suggest this method might not be optimal for parameter estimation at the individual level. A similar behavior could be seen with *V*_T_ estimates from SA. This variability was especially true for regions of small *V*_T_ (low-density regions). This observation is in agreement with a previous report of a VOI analysis, where we found the difference between 1TCM and 2TCM *V*_T_ values to be larger for cerebellum, medulla, and pons [16]. Indeed, model differences seemed to be a function of receptor density and consequently, a function of the underlying tissue configuration. As shown in our previous study [18], a 1TCM configuration leads to major errors in parameter estimation in low-binding regions, both from plasma input models and reference models relying on fast (1 T) kinetics for the reference tissue. On the other hand, such a range of differences might not be relevant for most study designs, as regions with low-density of receptors are generally not the main focus of an analysis. Nonetheless, even though this region-dependent range of agreement was seen in all three methods, the range of differences to 2TCM values was smallest for Logan, which does not assume a specific compartmental configuration (Fig. 3). As a consequence, despite the significant bias compared to reference values, Logan can be considered the most robust of the three methods for the generation of parametric *V*_T_ images, showing a higher precision independent of receptor density levels. Yet, it is important to note that while VOI analyses were performed with a fixed blood volume of 5 %, parametric methods either used it as a fit parameter or freely incorporated its contribution into the model. Although a limitation, previous work has shown that different contributions of blood to the overall signal did not significantly affect the main outcome parameter [18].

Parametric images of receptor binding were also comparable between different parametric methods. Moreover, a good correspondence to VOI-counterparts was seen for all reference methods, with high *R* ^2^ values and only small over- and underestimations seen for RPM and SRTM2, respectively (Table 3). In comparison with reference 2TCM values, correlation remained high for all methods, and linear regression slopes were closer to the identity line for the SRTM2. However, RLogan consistently underestimated binding, with a regression slope of 0.86. Interestingly, while RPM and SRTM2 had overall bias similar to those of RLogan (−0.10, 0.05, and −0.17, respectively), the range of differences to 2TCM values was larger for those methods (Fig. 3). In fact, the differences seen between SRTM2 and 2TCM estimates reached 0.5 for regions of low-binding—values higher than the actual *BP*_ND_ estimates—translating into relative errors of more than 100 % (Fig. 4b, c). However, those correspond to *BP*_ND_ values close to zero, where large relative errors are not surprising, nor meaningful. On the other hand, it is also important to notice that all reference-based methods underestimate the true *BP*_ND_ from the start, due to the considerable levels of specific binding present in the rat pons [16]. Nonetheless, RLogan displayed the highest precision in estimating receptor binding, as can be seen in Fig. 3. Therefore, despite its bias to 2TCM values, RLogan can be considered the most robust reference-based parametric method for the generation of receptor binding images.

While *V*_T_ and *BP*_ND_ are important kinetic parameters, both require dynamic scanning for their estimation. As a consequence, SUV and SUVR determined from static late images are widely applied for both regional and parametric analyses. While SUV does not necessarily correspond to *V*_T_, both parameters are directly related. This was also seen in our findings, where good correlation (*R* ^2^ > 0.95) was found between parametric SUV determined at three p.i. intervals and 2TCM *V*_T_ (Fig. 5a). In turn, SUVR is closely related to *BP*_ND_, and when equilibrium is reached, SUVR–1 is expected to correspond to DVR–1. Therefore, a more direct comparison between SUVR–1 from different intervals p.i. and *BP*_ND_ could be performed. However, as can be seen in Fig. 1, this method overestimated *BP*_ND_ from other methods and DVR–1 from 2TCM (Table 3). The largest overestimation of 2TCM DVR–1 was seen for SUVR_40–50_ and the smallest for SUVR_50–60_, which might indicate equilibrium is not reached at earlier intervals. Nonetheless, all SUVR–1 strongly correlated to 2TCM DVR–1. It is important to notice that despite the high correlations, the performance of SUV and SUVR–1 was region-dependent (Figs. 5a and 6a, respectively). However, when assessing agreement by a ratio Bland-Altman, the results suggested a scaling effect for SUV, since relative errors were similar across regions (Fig. 5b). Although useful, SUV and SUVR images must be applied carefully in group comparisons, interventional and longitudinal studies, since they are especially affected by changes in metabolism and tissue perfusion [26].

The results of this study indicate that parametric analysis of [^11^C]flumazenil images of the rat brain can be satisfactorily performed, and, as a consequence, parametric imaging and SPM analysis would allow direct statistical comparison across groups and/or conditions without prior assumptions on regions of interest. In fact, SPM analysis has previously been able to detect more subtle disease-related changes in an animal model of inflammation imaged with [^11^C]PK11195, for example [27]. However, the homogeneous dataset analyzed in this study did not allow for a similar assessment of the performance of parametric imaging in group comparison nor was this within the scope of the study. Thus, further studies should be performed in order to evaluate the benefit of parametric imaging of [^11^C]flumazenil binding in the rat brain for different conditions and study designs. In general, since Logan and RLogan outperformed other methods in precision and are of robust and simple implementation, both methods are preferred for group comparisons and longitudinal studies. On the other hand, alternatives such as BFM or RPM can be of value in generating parametric images with additional information (*K*_1_ or *R*_1_), while SUV and SUVR images could be useful when dynamic scanning is not possible, despite under- and overestimation of kinetic parameters.

## Conclusion

This study demonstrated several parametric methods performed well for the generation of [^11^C]flumazenil distribution and binding images, with a good correlation to 2TCM-derived values being observed across all methods. Therefore, a specific method should be applied according to what information is of interest to a specific research question. Nonetheless, the most precise and straightforward methods were found to be Logan and RLogan, while BFM or RPM could be considered when information on perfusion is of significance.
